# Role of DNA Repair Pathways in Response to Zidovudine-induced DNA Damage in Immortalized Human Liver THLE2 Cells

**Published:** 2013-03

**Authors:** Qiangen Wu, Frederick A. Beland, Ching-Wei Chang, Jia-Long Fang

**Affiliations:** 1 *Division of Biochemical Toxicology, National Center for Toxicological Research, U.S. Food and Drug Administration, Jefferson, AR 72079, USA;*; 2 *Division of Bioinformatics and Biostatistics, National Center for Toxicological Research, U.S. Food and Drug Administration, Jefferson, AR 72079, USA;*; 3 *Department of Environmental Health, Indiana University, Bloomington, IN 47405, USA*

**Keywords:** DNA damage and repair, nucleotide excision repair, THLE2 cells, zidovudine

## Abstract

The nucleoside reverse transcriptase inhibitor zidovudine (3’-azido-3’-dexoythymidine, AZT) can be incorporated into DNA and cause DNA damage. Previously, we determined that the human hepatocellular carcinoma HepG2 cells are more susceptible to AZT-induced toxicities than the immortalized normal human liver THLE2 cells and the nucleotide excision repair (NER) pathway plays an essential role in the response to AZT-induced DNA damage. We have now investigated if the effects of AZT treatment on the expression levels of genes related to DNA damage and repair pathways contribute to the differences in sensitivity to AZT treatment between HepG2 cells and THLE2 cells. Of total 84 genes related to DNA damage and repair, two, five, and six genes were up-regulated more than 1.5-fold at 50, 500, and 2,500 µM AZT groups compared with that of control THLE2 cells. Seven genes showed a decreased expression of more than 1.5-fold following the 2,500 µM AZT treatment. Two-sided multivariate analysis of variance indicated that the change in expression of genes involved in apoptosis, cell cycle, and DNA repair pathways was significant only at 2,500 µM AZT. Statistically significant dose-related increases were identified in *XPC* gene expression and GTF2H1 protein level after the AZT treatments, which implicated the NER pathway in response to the DNA damage induced by AZT. In contrast, AZT treatment did not alter significantly the expression of the *APE1* gene or the levels of APE1 protein. These results indicate that the NER repair pathway is involved in AZT-induced DNA damage response in immortalized human hepatic THLE2 cells.

## INTRODUCTION

Zidovudine (3’-azido-3’-dexoythymidine, AZT) was the first nucleoside reverse transcriptase inhibitor approved by the U.S. Food and Drug Administration for the treatment of HIV-1/AIDS. Since then, tremendous advances have been made in the treatment of AIDS patients and the prevention of mother-to-child transmission of HIV-1 by combining the administration of AZT with other anti-HIV drugs (1). However, concerns over both acute toxicities and long-term health risks have arisen due to the possible genotoxicity of AZT to drug-exposed mothers and their fetuses.

AZT is tumorigenic in animal models. An increased risk of vaginal epithelium tumors has been demonstrated in adult mice and rats after chronic exposure (2-4), and mice exposed to AZT transplacentally have increased incidences of tumors in a number of organs including liver, lung, mammary gland, and ovaries (5-8). It is believed the specific chemical structure of AZT contributes to its carcinogenesis. As an analogue of thymidine, AZT can be phosphorylated and incorporated into host DNA in place of thymidine and cause damage to the DNA. Recent studies have demonstrated genotoxic manifestations of AZT, including the induction of micronuclei, sister chromatid exchanges, chromosomal aberrations, mitochondrial damage, decreases in telomere length, centrosomal abnormalities, and nuclear bud formation (9-17).

DNA damage checkpoints induced by AZT may lead to cell cycle arrest and DNA repair (18-20). Cooper and Lovett (21) have demonstrated that exonuclease III, but not DNA polymerases, plays a major role in the removal of AZT-monophosphate from *E. coli* DNA. It also has been reported that a 3’-5’ exonuclease is involved in the removal of AZT incorporated into DNA of human leukemic cells *in vitro* (22, 23). Furthermore, we found that XPC (complementation group C), a component of the nucleotide excision repair (NER) pathway, is essential for the repair of AZT-induced DNA damage in human hepatoma HepG2 cells (24). Although there have been a number of studies to investigate DNA repair mechanisms of AZT in carcinoma cell lines and bacteria (21, 24-26), little is known about this machinery’s response to AZT in normal human cells. We have demonstrated previously that human hepatoma HepG2 cells are more sensitive than immortalized normal human hepatic THLE2 cells to AZT treatment as indicated by an inhibition of cell growth in the THLE2 cells with much higher concentrations being required (18). As a continuation of our studies to evaluate the potential human cancer risk after long term AZT exposure, we have now investigated if the effects of AZT treatment on the expression levels of genes related to DNA damage and repair pathways contribute to the differences in sensitivity to AZT treatment between HepG2 cells and THLE2 cells.

## MATERIALS AND METHODS

### Chemicals and antibodies

AZT was purchased from Cipla Ltd. (Mumbai, India). Penicillin-streptomycin and 2.5% trypsin were purchased from Fisher Scientific (Pittsburgh, PA). Dulbecco’s phosphate buffered saline (PBS), epidermal growth factor, LHC-8 medium, and SuperScript III first-strand synthesis kits were obtained from Invitrogen Life Technologies (Carlsbad, CA). Fetal bovine serum was acquired from Atlanta Biologicals (Lawrenceville, GA). RNeasy Mini kits and RT^2^ Profile PCR arrays for DNA damage and repair were purchased from Qiagen Sciences (Germantown, MD). The BCA Protein Assay kit and RIPA buffer were obtained from Pierce Biotechnology (Rockford, IL). Complete protease inhibitor cocktail was purchased from Roche Applied Science (Mannheim, Germany). Antibodies to XPC, XPA, RPA1, ERCC1, and APE1 (apurinic/apyrimidinic endonuclease) were purchased from Santa Cruz Biotechnology (Santa Cruz, CA). The antibody to GTF2H1 (general transcription factor IIH subunit 1) was obtained from Abcam Inc. (Cambridge, MA).

### Cell culture and treatments

SV40 large T antigen-immortalized normal human liver epithelial cells (THLE2) (American Type Culture Collection, Manassas, VA) were cultured in LHC-8 medium supplemented with 70 ng/ml phosphoethanolamine, 5 ng/ml epidermal growth factor, 10% fetal bovine serum, and antibiotics at 37°C in a humidified atmosphere containing 95% air and 5% CO_2_. AZT stock solution (25 mM) was prepared in THLE2 complete culture medium. The cells were treated with 50, 500, or 2,500 µM AZT for two weeks. The 2-week exposure gave the maximum response in THLE2 cells treated with AZT based upon our previous experiments (18). Control cells were fed complete culture medium free of AZT. Each of the incubations was performed in triplicate.

### Pathway-specific real-time PCR array

At the end of the AZT treatments, total RNA was isolated from the treated and control cells using RNeasy Mini kits. First strand cDNA synthesis was conducted with SuperScript III first-strand synthesis system for RT-PCR according to the manufacturer’s protocol. Subsequently, a human DNA damage signaling RT^2^ Profile PCR array was used to determine the effect of AZT treatment on the expression of 84 genes with known roles in the response to and repair of DNA damage. The functional gene groupings in the human DNA damage signaling pathway PCR Array are shown in Table 1. Real-time PCR was performed in triplicate for each sample using a Bio-Rad iCycler Real-Time PCR system following the manufacturer’s instructions. The Ct values for the 84 genes were normalized to five housekeeping genes (*B2M*, *HPRT1*, *RPL13A*, *GAPDH*, and *ACTB*). Comparisons between AZT treated groups and the controls were conducted using the ΔΔCt method to assess the relative changes in gene expression levels.

**Table 1:** Functional gene groupings in the human DNA damage signaling pathway PCR array

Gene group	Subgroup	Genes

Apoptosis		*ABL1, BRCA1, CIDEA, GADD45A, GADD45G, GML, IHPK3, PCBP4, AIFM1 (PDCD8), PPP1R15A, RAD21, TP53, TP73.*
Cell cycle		*ATR, BRCA1, CHEK1, CHEK2, DDIT3 (CHOP), FANCG, GADD45A, GML, GTSE1, HUS1, MAP2K6, MAPK12, NBN (NBS1), PCBP4, PPP1R15A, RAD17, RAD9A, RAD1, RBBP8, SESN1, SMC1A (SMC1L1), TP53, ZAK,*
DNA repair	Damaged DNA binding	*ANKRD17, BRCA1, DDB1, DMC1, ERCC1, FANCG, FEN1, MPG, MSH2, MSH3, N4BP2, NBN (NBS1), OGG1, PMS2L3 (PMS2L9), PNKP, RAD1, RAD18, RAD51, RAD51L1, REV1 (REV1L), SEMA4A, XPA, XPC, XRCC1, XRCC2, XRCC3.*
	Base excision repair	*APEX1, MBD4, MPG, MUTYH, NTHL1, OGG1, UNG.*
	Double-strand break repair	*CIB1, FEN1, XRCC6 (G22P1), XRCC6BP1 (KUB3), MRE11A, NBN (NBS1), PRKDC, RAD21, RAD50.*
	Mismatch repair	*ABL1, ANKRD17, EXO1, MLH1, MLH3, MSH2, MSH3, MUTYH, N4BP2, PMS1, PMS2, PMS2L3 (PMS2L9), TP73, TREX1*
	Nucleotide excision repair	*XPC, XPA, RPA1, GTF2H1, ERCC1*
	Others	*APEX2, ATM, ATRX, BTG2, CCNH, CDK7, CRY1, PCNA ERCC2 (XPD), GTF2H2, IGHMBP2, LIG1, MNAT1, SUMO1.*

### Western blotting

At the end of the AZT treatment, the cells were trypsinized and washed three times in PBS. Forty µl of cell pellets were lysed in five volumes of RIPA buffer supplemented with complete protease inhibitor cocktail. After incubation on ice for 30 min, followed by sonication for 30 sec with a 50% pulse, the supernatants were collected by centrifugation at 14,000 g for 20 min and stored in aliquots at −65°C until analysis. The amount of protein in the cell lysates was determined using a BCA Protein assay kit. Forty µg of cell lysates were separated by 12% SDS-polyacrylamide gel electrophoresis and electrophoretically transferred onto PVDF membranes. The blots were blocked with 5% milk and probed with anti-XPC, XPA, RPA1, GTF2H1, ERCC1, and APE1 antibodies, followed by a secondary antibody conjugated to horseradish peroxisdase. The levels of β-actin were detected in the same manner and used as a loading control. The intensity of each band was quantified with ImageJ software (NIH, Bethesda, MD).

### Statistical analyses

Data are presented as mean ± SD. Two-sided multivariate analysis of variance (MANOVA) was used to assess the significance of increases in the expression of pathways from real-time PCR array data (27). Dose-related trends were investigated by regression analysis. One-way analysis of variance followed by pairwise-comparisons using Dunnett’s test was used to determine the significance between specific AZT treatments and control cells. Data were logarithm transformed if they failed variance homogeneity tests. The difference was considered statistically significant when the *P* value was less than 0.05.

## RESULTS

### Expression of DNA damage and repair related genes after AZT treatment

The effects of a two-week AZT treatment on the expression profile of 84 genes related to DNA damage and repair were investigated in THLE2 cells. After the treatment, two (2.4%), five (6.0%), and six genes (7.1%) were up-regulated more than 1.5-fold after the 50, 500, and 2,500 µM AZT treatment (Table 2). Up-regulation of polynucleotide kinase 3’-phosphatase (*PNKP*) was present in all AZT treatment groups. Only two of the 84 genes were up-regulated more than 2-fold, and this occurred with only 2,500 µM AZT. These genes were *BTG2* and *PCBP4*, which showed increased expressions of 2.1- and 2.2-fold. As shown in Table 2, decreased gene expression (>1.5-fold) was only observed at 2,500 µM AZT, and this occurred with seven genes (8.3%). None of the genes was down-regulated greater than 2-fold.

**Table 2:** Fold change of genes related to DNA damage and repair signaling pathway in THLE2 cells treated with AZT for two weeks

	Fold change	50 μM AZT		500 μM AZT		2,500 μM AZT	
		N	%	N	%	N	%

Up-regulation	>1.5	2	2.4	5	6.0	6	7.1
Down-regulation	>1.5	0	0	0	0	7	8.3

### Pathway based analysis on DNA damage and repair genes expression after AZT treatment

Of the 84 genes on the DNA damage signaling pathway PCR array, 13 genes are directly associated with apoptosis, 23 with cell cycle progression regulation, and 52 with DNA repair (Table 1). MANOVA analysis indicated that the change in the expression of the genes involved in apoptosis, cell cycle progression regulation, and DNA repair pathways, was significant only at 2,500 µM AZT (Table 3). The DNA repair pathway genes were further subdivided into groups including damaged DNA binding, base excision repair (BER), double strand break repair, mismatch repair, and NER. MANOVA analysis indicated that none of the subgroups had a significantly increased or decreased gene expression at any dose of AZT (Table 3).

**Table 3:** Analysis of DNA repair pathways, as assessed by MANOVA, in THLE2 cells

Gene set	Subgroup pathway	Number of Gene	AZT (*P* value^a^)		
			50 μM	500 μM	2,500 μM

Apoptosis		13	0.935	0.284	**0.019**
Cell cycle		23	0.525	0.153	**0.012**
DNA repair		52	0.776	0.684	**0.019**
	Damaged DNA binding	26	0.247	0.086	0.093
	Base-excision repair	7	0.800	0.811	0.894
	Double-strand break repair	9	0.563	0.333	0.210
	Mismatch repair	14	0.671	0.185	0.312
	Nucleotide excision repair	5	0.793	0.801	0.104

a Compared with that of the control cells. Values in bold are significant at *P*<0.05.

### Relative gene expression of NER subpathway in AZT-treated THLE2 cells

**Figure 1:**
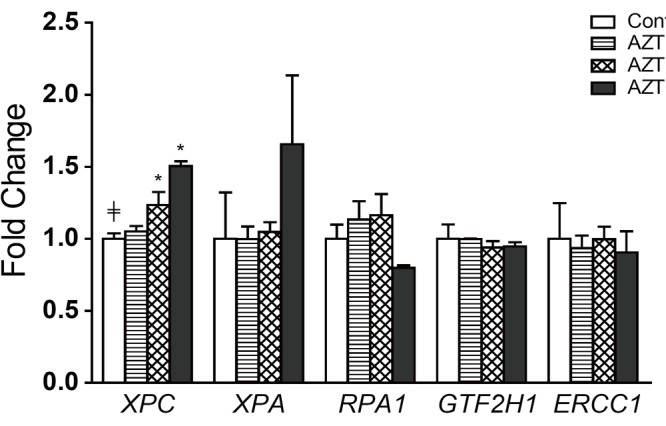
Relative gene expression levels of NER pathway in THLE2 cells after AZT treatment. Data are expressed as the mean ± SD from three independent experiments. ≠, significant (*P*<0.05) dose-related trend; *Significantly (*P*<0.05) different from the control group

Our previous study indicated that the NER pathway was the primary repair machinery in the repair of AZT-induced DNA damage in human hepatoma HepG2 cells (24). The relative level expression of five genes involved in NER pathway (*i.e.,* *XPC*, *XPA*, *RPA1*, *GTF2H1*, and *ERCC1*) was further analyzed in THLE2 cells after two weeks of AZT treatment. As depicted in Figure 1, of the five genes investigated, only the expression of *XPC* was increased in a dose-related manner, with the extent of the increase being significant at 500 and 2,500 µM AZT (1.2- and 1.5-fold, respectively).

### NER protein expression in THLE2 cells treated with AZT for 2 weeks

**Figure 2:**
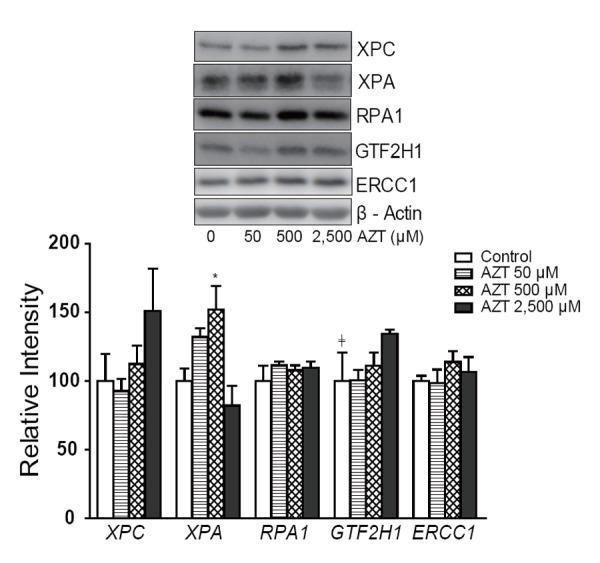
NER protein expression levels in THLE2 cells after AZT treatment. Data are expressed as the mean ± SD from three independent experiments. ≠, significant (*P*<0.05) dose-related trend; *Significantly (*P*<0.05) different from the control group

The protein levels of XPC, XPA, RPA1, GTF2H1, and ERCC1 in THLE2 cells after two weeks of AZT treatment are shown in Figure 2. Although there appeared to be an increase in XPC, especially at 2,500 µM AZT, the increase did not reach statistical significance. There was an increase in the levels of XPA, but this occurred at only 500 µM AZT. In addition, an increasing dose trend was observed with GTF2H1.

### APE1 gene and protein expression in AZT-treated THLE2 cells

**Figure 3:**
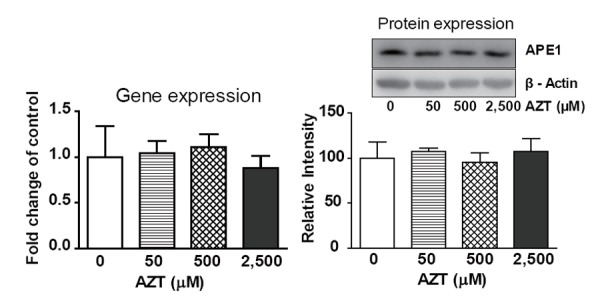
*APE1* gene expression and APE1 protein levels in THLE2 cells treated with AZT for two weeks. Data are expressed as the mean ± SD from three independent experiments

The gene expression and protein levels of APE1, a key repair factor in the BER pathway, were investigated in THLE2 cells after exposure to AZT for two weeks. As shown in Figure 3, neither gene expression nor protein levels showed significant changes compared with that of control.

## DISCUSSION

In this study, we have investigated the effects of AZT treatment on the expression levels of genes related to DNA damage and repair pathways in normal human hepatic THLE2 cells. Out of total 84 genes related to DNA damage and repair, 2,500 µM AZT treatments caused six genes to be up-regulated and seven genes to be down-regulated when compared to the control cells. MANOVA analysis indicated that the expression of genes involved in the apoptosis, cell cycle, and DNA repair pathways was significantly increased only at 2,500 µM AZT. Dose-related increases were identified in *XPC* gene expression and GTF2H1 protein levels, components that are involved primarily in the NER pathway after AZT treatment. These results were consistent with our previous findings in hepatoma HepG2 cells after AZT treatment and indicate that NER is involved in AZT-induced DNA damage response in not only human hepatoma cells but also immortalized normal human liver epithelial cells (24). However, a comparison of the number of genes affected and the change in expression levels of the affected genes and proteins indicates that the DNA repair response of THLE2 cells to AZT-induced DNA damage was less than that observed in hepatoma HepG2 cells (24). For example, incubation of HepG2 cells with 100 μM AZT resulted in the up-regulation of 22 of 84 genes associated with DNA damage and repair (24), whereas in THLE2 cells treated with 500 μM AZT only 5 of 84 genes related to DNA damage and repair were up-regulated (Table 2).

AZT was originally synthesized in the 1960s as a possible anti-cancer agent (11). Treatment of cells with varying concentrations of AZT induced inhibition of cell growth and apoptosis in three human melanoma cell lines without affecting the growth of non-tumorigenic cells (20). In our previous studies, the immortalized normal human liver THLE2 cells also showed a greater resistance than the hepatoma HepG2 cells to the toxicity of AZT (18). The insensitivity of THLE2 cells to AZT treatment appears to be due to the fact that these normal human hepatic cells express phenotypic characteristics of normal adult liver epithelial cells and retain phase I and II enzyme activities (28). Thymidine kinase 1 (TK1) catalyzes the monophosphorylation of AZT in the pathway of activation to DNA incorporation and is considered to be the rate-limiting step in the metabolism of AZT. It has been reported that cells may become resistant to AZT or cross-resistant to thymidine and deoxycytidine analogs partially through inactivation of TK1 after long term AZT exposure (29-34). TK1 activity and its protein expression levels were decreased following AZT treatment in THLE2 cells (data not shown), and such a decrease in TK1 would, at least partially, contribute to the resistance of THLE2 cells to AZT treatment.

We identified a significant difference in the apoptosis and cell cycle gene groups between the cells treated with 2,500 µM AZT and the control cells (Table 3). It is well documented that AZT induces apoptosis in a variety of cells including mouse skeletal muscle cells (16), mouse myocardium (35), human adipocytes (36), human liver HepG2 and THLE2 cells (18, 24), and human parathyroid cancer cells (26). Additionally, cell cycle perturbations as a consequence of AZT induced DNA damage have been observed in mouse NIH 3T3 cells (19), human hepatoma HepG2 cells and normal hepatic THLE2 cells (18), human chronic myeloid leukemia K562 cells (37), human promyelocytic HL60 cells (38), human colon carcinoma WiDr cells (39), human T lymphoid CCRF-CEM cells (40), and human melanoma cell lines (518A2, A375, and Mel Juso) (20). The genes increased more than two fold in the current study were *BTG2* and *PCBP4*, genes that play a critical role in cell cycle control and cellular response to DNA damage. *BTG2* is involved in the regulation of the G1/S transition of the cell cycle by inhibiting pRb function and is critical for normal cell growth and proliferation (41, 42). *PCBP4* is induced by the p53 tumor suppressor, and the encoded protein can suppress cell proliferation by inducing apoptosis and cell cycle arrest in G2/M (43, 44). Several studies have indicated that the presence of DNA damage induced by AZT can lead to cell cycle checkpoint arrest to allow for DNA repair (18-20). The cell cycle arrest in THLE2 cells (18) highlights the possible role of cell cycle arrest for DNA repair and cell survival after AZT exposure. The activation of a checkpoint pathway that arrests the cell cycle to permit DNA repair and the fact that AZT effects were partially or totally restored during the recovery period suggests that there was active removal of AZT from DNA (18).

There have been relatively few studies investigating the possible role of DNA repair in the removal of AZT from DNA. The kinetics of DNA rejoining, considered to be an indicator of DNA repair, revealed that AZT-induced DNA breaks are repaired slowly in human colonic CaCo-2 cells and diploid lung fibroblast HEL cells (25). In *E. coli,* Cooper and Lovett demonstrated that exonuclease III but not DNA polymerases play a major role in the removal of AZT-monophosphate from DNA (21). Based upon this observation the authors predicted that the APE1 would play a role in the tolerance of human cells to AZT; however, at neither the gene nor protein expression levels did APE1 show any significant change in THLE2 cells after a two-week AZT treatment. Previously, we demonstrated that XPC, via the NER pathway, is essential for the repair of AZT-induced DNA damage in human hepatoma HepG2 cells (24). In the current study, the expression of *XPC* and level of GTF2H1 were increased in a dose-related manner in AZT treated THLE2 cells. In addition, a protective role of the NER pathway against aneuploidy and centrosomal aberrations induced by AZT treatment has been demonstrated in a transgenic mouse model (17, 45). These observations are consistent with our hypothesis that the NER pathway is the primary mechanism to remove AZT incorporated into the DNA of both malignant and immortalized normal human hepatocytes.

## FUNDING

This research was supported through an interagency agreement between the National Center for Toxicological Research, U.S. Food and Drug Administration, and the National Toxicology Program, National Institute of Environmental Health Sciences (NCTR/FDA IAG: 224-12-0003; NIH AES12013).
